# A simple method to increase the proportion of bone marrow-derived macrophages positive for M-CSFR using the reducing agent dithiothreitol (DTT)

**DOI:** 10.1016/j.mex.2018.11.014

**Published:** 2018-11-27

**Authors:** Ryota Hashimoto, Hiroyuki Daida, Takao Okada, Youichi Katoh

**Affiliations:** a Department of Physiology, Juntendo University Faculty of Medicine, Hongo 2-1-1, Bunkyo-ku, Tokyo, 113-8421, Japan; b Department of Cardiology, Juntendo University Graduate School of Medicine, Hongo 2-1-1, Bunkyo-ku, Tokyo, 113-8421, Japan; c Juntendo University, Faculty of International Liberal Arts, Hongo 2-1-1, Bunkyo-ku, Tokyo, 112-8421, Japan

**Keywords:** Method to increase the proportion of macrophages positive for M-CSFR, Bone marrow-derived macrophages, Macrophage colony-stimulating factor receptor (M-CSFR), Dithiothreitol (DTT), Lipopolysaccharide (LPS), Atherosclerosis

## Abstract

Only a few bone marrow-derived macrophages (BM-MΦ) are positive for macrophage colony-stimulating factor receptor (M-CSFR). Thus, a method is needed to increase the proportion of BM-MΦ that are positive for M-CSFR to facilitate the investigation of the effects of M-CSFR downregulation on various diseases. We used mouse primary BM-MΦ to evaluate the expression of M-CSFR on the cytoplasmic membrane using flow cytometry. Treatment with a reducing agent, dithiothreitol (DTT), increased the proportion of BM-MΦ that were positive for M-CSFR, and this increase was time dependent. We next determined whether DTT-treated BM-MΦ can lead to the downregulation of M-CSFR. Treatment with lipopolysaccharide (LPS) for 24 h. decreased the proportion of DTT-treated BM-MΦ that were positive for M-CSFR. These results suggest that DTT treatment increases the proportion of BM-MΦ that are positive for M-CSFR and that the upregulation of M-CSFR on BM-MΦ can be abrogated by treatment with LPS. Here, we propose a simple method to increase the number of M-CSFR-positive BM-MΦ using the reducing agent DTT, which could be useful in investigations of the relationship between the downregulation of M-CSFR and some diseases.

• The proportion of BM-MΦ that expresses M-CSFR on the membrane increases by approximately twice following DTT treatment.

## Graphical abstract

**Figure:**
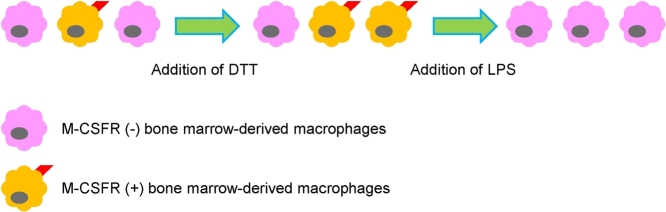

**Specifications Table**

**Table:** 

Subject Area	*Biochemistry, Genetics and Molecular Biology*
More specific subject area:	Upregulation and downregulation
Method name:	Method to increase the proportion of macrophages positive for M-CSFR
Name and reference of original method:	None
Resource availability:	None

## Method details

**Fig. 1:**
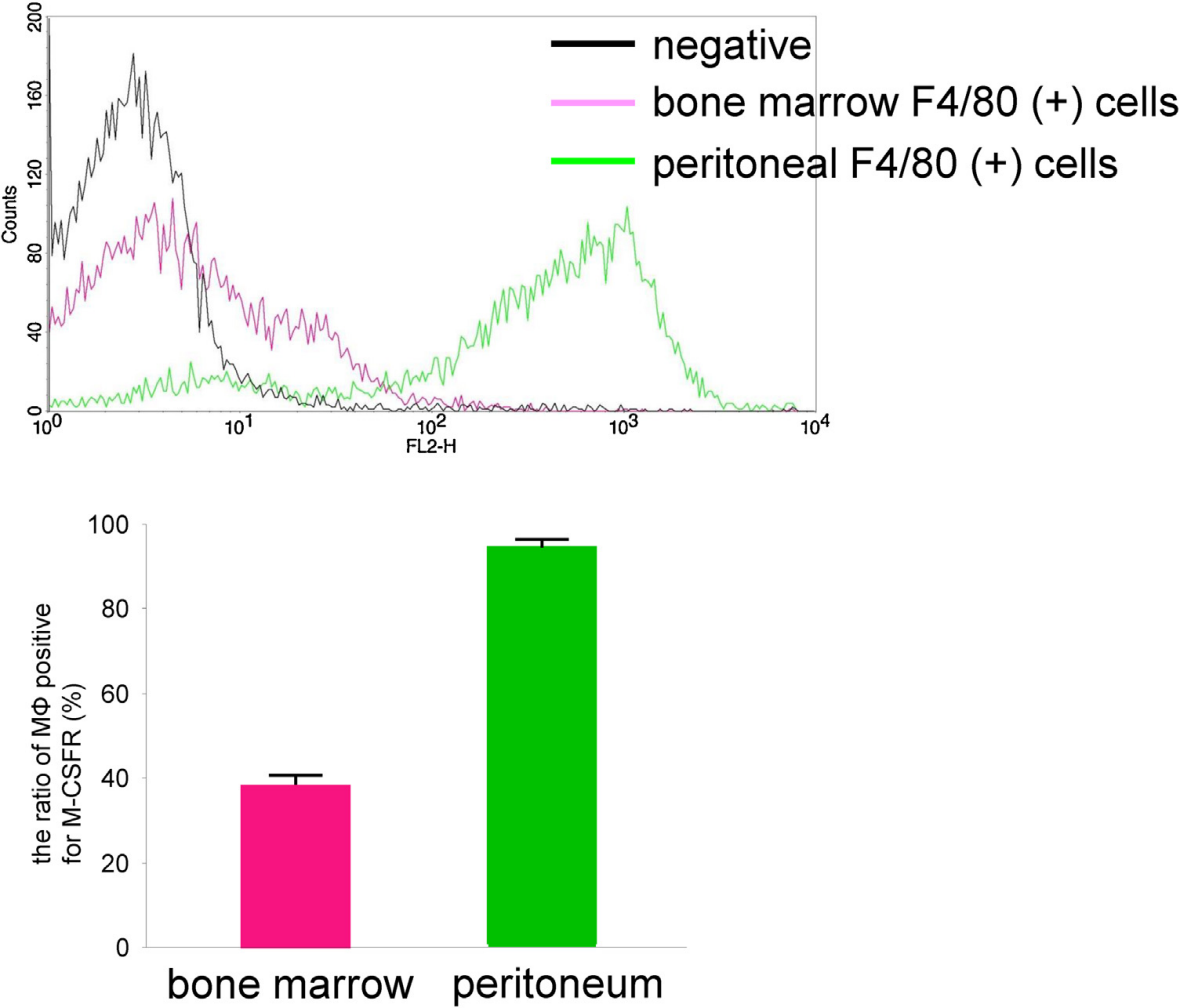
Proportion of peritoneal macrophages and BM-MΦ that is positive for M-CSFR. Isolated bone marrow cells and isolated peritoneal cells were stained with phycoerythrin (PE)-conjugated anti-macrophage colony-stimulating factor receptor (M-CSFR) antibody and fluorescein isothiocyanate (FITC)-conjugated anti-F4/80 antibody and were analyzed by flow cytometry. (Upper) Representative histograms for bone marrow macrophages (BM-MΦ, F4/80 positive bone marrow cells) and peritoneal macrophages (F4/80 positive peritoneal cells) are shown. (Bottom) The proportion of macrophages that are positive for M-CSFR (%) is shown. n = 5.

Most experimental studies have been performed on macrophages derived from the spleen, peritoneum, and bone marrow. It has been reported that only 2.4 ± 0.4% of spleen-derived cultured macrophages, 3.6 ± 0.2% of peritoneal cultured macrophages, and 65.4 ± 3.0% of bone marrow (BM)-cultured macrophages (MΦ) express macrophage colony-stimulating factor receptor (M-CSFR, CSF-1R, c-fms, CD115) on the plasma membrane [1], indicating that only a small proportion of macrophages is positive for M-CSFR. In our experiment, 95.3 ± 0.8% of isolated peritoneal macrophages expressed M-CSFR and 37.8 ± 2.7% of isolated BM-MΦ expressed M-CSFR (Fig. 1). Thus, we have used BM-cultured MΦ and explored a method to increase the number of M-CSFR-positive macrophages. The use of this proposed simple method allows the increase in the number of M-CSFR-positive BM-MΦ on the plasma membrane.

Procedure: cell culture of primary bone marrow adherent cells [[2], [3], [4]]

1 Male C57Bl/6 mice (Charles River Laboratories Japan, Inc., Kanagawa, Japan) were euthanized by cervical dislocation, and bone marrow cells were collected from the tibia and femur.
2 Bone marrow cells were cultured in RPMI 1640 (Invitrogen, New York, USA) containing 20% fetal bovine serum (FBS; Equitech-Bio, Texas, USA) at 37 °C in 5% CO_2_/95% air for 14 days.
3 We selectively maintained adherent cells by removing floating cells during the change in medium.

Bone marrow adherent cells that take up Ac-LDL are macrophages.

Macrophages, endothelial cells, and endothelial progenitor cells are known to take up modified LDL [5]. We have reported that most of the bone marrow adherent cells that participated in the uptake of acetylated low-density lipoprotein (Ac-LDL) under our culture conditions are macrophages [6].

Procedure: measurement of M-CSFR expression on BM-MΦ

**Fig. 2:**
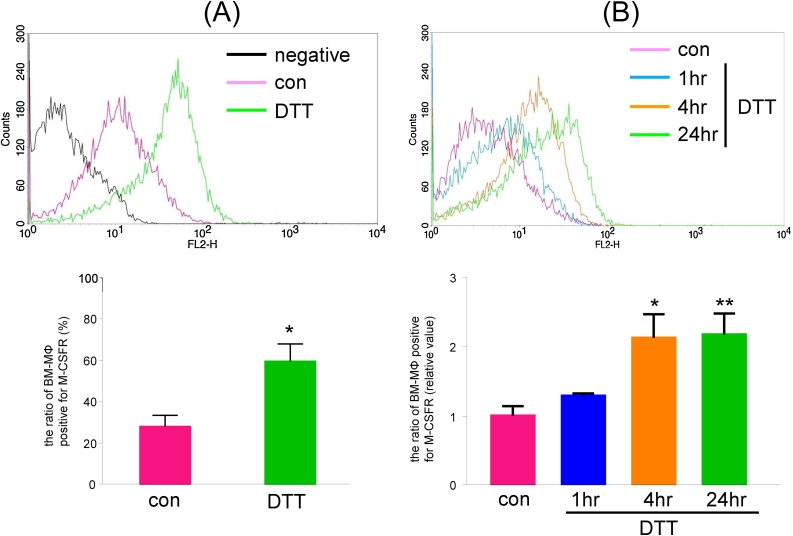
DTT treatment increases the proportion of BM-MΦ that are positive for M-CSFR. BM-cultured cells were treated with vehicle or the reducing agent dithiothreitol (DTT, 1 mM) for 1 h, 4 h, or 24 h and then were incubated with 400 ng/mL of acetylated-low density lipoprotein (Ac-LDL) labeled with 3,3′-dioctadecyloxacarbocyanine perchlorate (Dio). After 4 h, the cells were stained with PE-conjugated anti-M-CSFR antibody and were analyzed by flow cytometry. (Upper) Representative histograms for BM-MΦ (bone marrow cells that took up Ac-LDL) treated with DTT are shown. (Bottom) The proportion of BM-MΦ that was positive for M-CSFR (A, %; B, relative value) is shown. (A) n = 5; (B) n = 3; * P < 0.05, ** P < 0.01 vs. control (vehicle).

1 Bone marrow adherent cells were treated with 400 ng/mL of Ac-LDL (Biomedical Technologies, Inc., Madrid, Spain) labeled with 3,3′-dioctadecyloxacarbocyanine perchlorate (Dio) for 4 h at 37 °C.
2 The cells were then detached, treated with FcR blocking reagent (Miltenyi Biotec GmbH, Bergisch Gladbach, Germany) for 10 min at 4 °C, and stained with the phycoerythrin (PE)-conjugated anti-M-CSFR antibody (Miltenyi Biotec) for 10 min at 4 °C.
3 The cells were washed and analyzed using a BD FACSCalibur instrument (Becton, Dickinson and Company, New Jersey, USA).
4 24.4 ± 4.6% of the BM-MΦ (bone marrow adherent cells that took up Ac-LDL) were positive for M-CSFR (Fig. 2A).

DTT treatment increases the proportion of BM-MΦ that are positive for M-CSFR.

Treatment with a reducing agent dithiothreitol (DTT, 1 mM) for 24 h increased the proportion of BM-MΦ that were positive for M-CSFR to 51.7 ± 7.3% (Fig. 2A). This increase following DTT treatment was time dependent (Fig. 2B, vehicle control; 1.0 ± 0.1, 1 h of DTT; 1.3 ± 0.0, 4 h of DTT; 2.1 ± 0.3, 24 h of DTT; 2.2 ± 0.3-fold increase). These results suggest that DTT treatment increases the proportion of BM-MΦ that express M-CSFR on the cytoplasmic membrane.

**Fig. 3:**
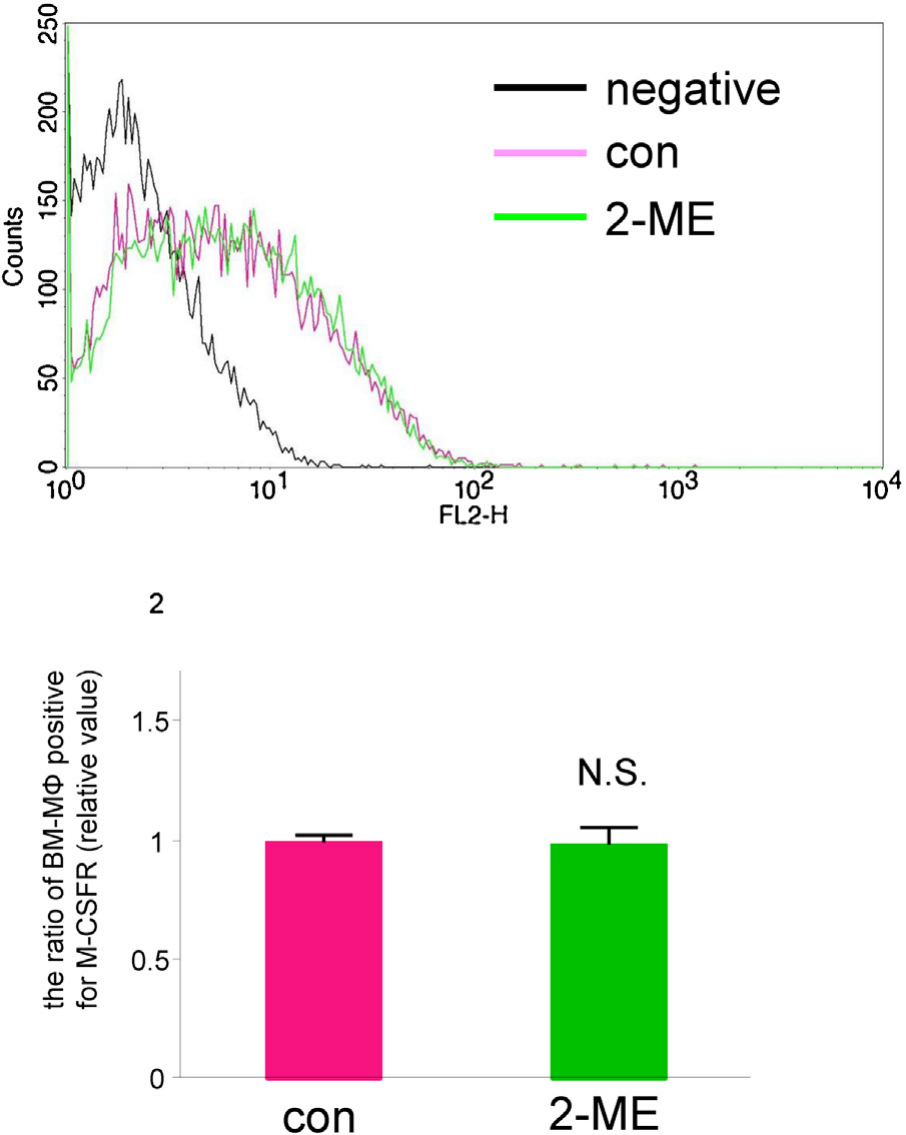
Treatment with a reducing agent did not alter the proportion of BM-MΦ that were positive for M-CSFR. BM-cultured cells were treated with vehicle or the reducing agent 2-mercaptoethanol (2-ME, 1 mM) for 24 h and then were incubated with 400 ng/mL of Dio-conjugated Ac-LDL for 4 h. The cells were stained with PE-conjugated anti-M-CSFR antibody and were analyzed by flow cytometry. (Upper) A representative histogram for BM-MΦ (bone marrow cells that took up Ac-LDL) treated with 2-mercaptoethanol is shown. (Bottom) The proportion of BM-MΦ that was positive for M-CSFR (relative value) is shown. n = 4 (N.S.: not significant).

**Fig. 4:**
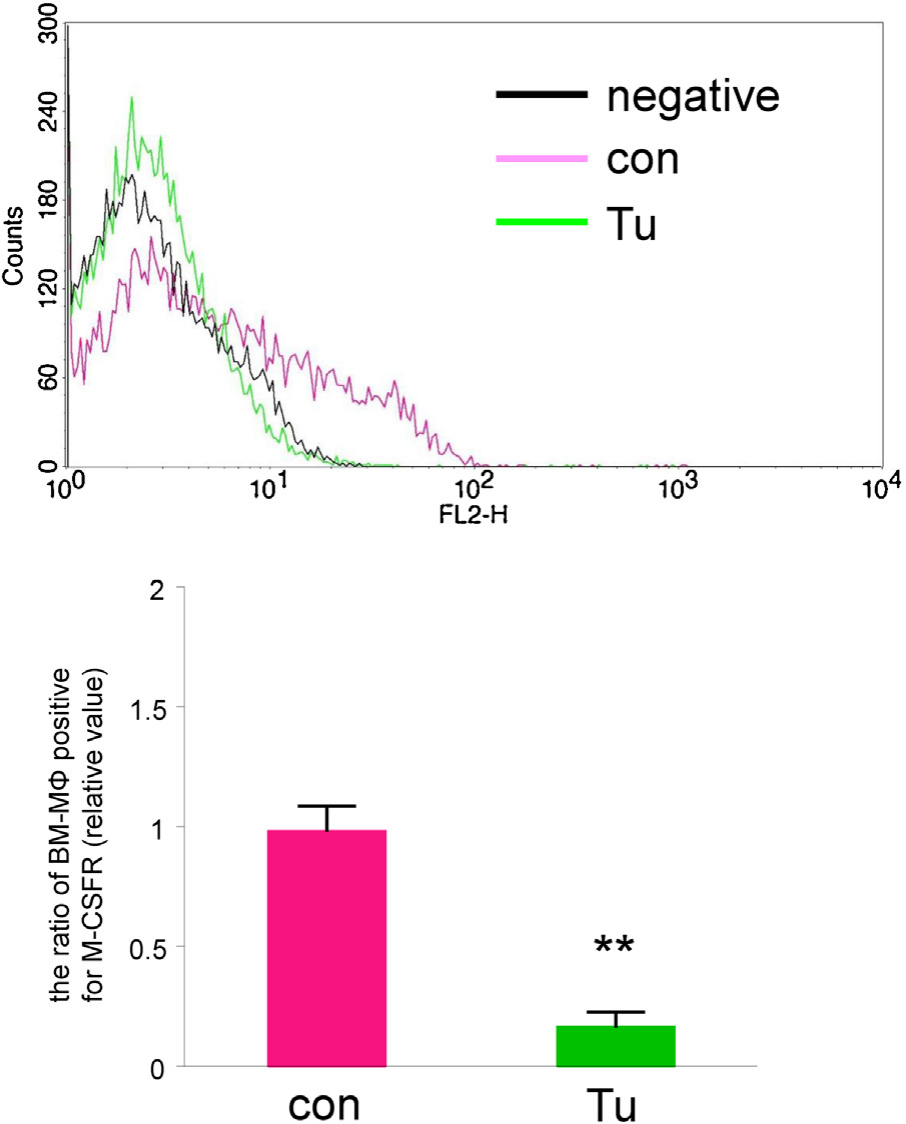
Treatment with an ER stress inducer decreases the proportion of BM-MΦ that is positive for M-CSFR. BM-cultured cells were treated with vehicle or the ER stress inducer tunicamycin (Tu, 0.5 mg/mL) for 24 h and then were incubated with 400 ng/mL of Dio-conjugated Ac-LDL for 4 h. The cells were stained with PE-conjugated anti-M-CSFR antibody and analyzed by flow cytometry. (Upper) A representative histogram for BM-MΦ (bone marrow cells that took up Ac-LDL) treated with tunicamycin is shown. (Bottom) The proportion of BM-MΦ that was positive for M-CSFR (relative value) is shown. n = 3 (** P < 0.01 vs. control (vehicle)).

DTT is known to be both a reducing reagent and an endoplasmic reticulum (ER) stress inducer. Treatment with another reducing agent, 2-mercaptoethanol (1 mM), for 24 h did not alter the proportion of BM-MΦ that were positive for M-CSFR (Fig. 3, vehicle control; 1.0 ± 0.0, 24 h of 2-mercaptoethanol; 0.99 ± 0.07-fold). Treatment for 24 h with a different ER stress inducer, tunicamycin (0.5 mg/mL), led to a decrease in the proportion of BM-MΦ that were positive for M-CSFR (Fig. 4, vehicle control; 1.0 ± 0.1, 24 h of tunicamycin; 0.17 ± 0.06-fold). These results suggest that DTT, but not a reducing reagent, or an ER stress inducer increases the proportion of BM-MΦ that are positive for M-CSFR.

DTT-treated BM-MΦ can lead to the downregulation of M-CSFR.

**Fig. 5:**
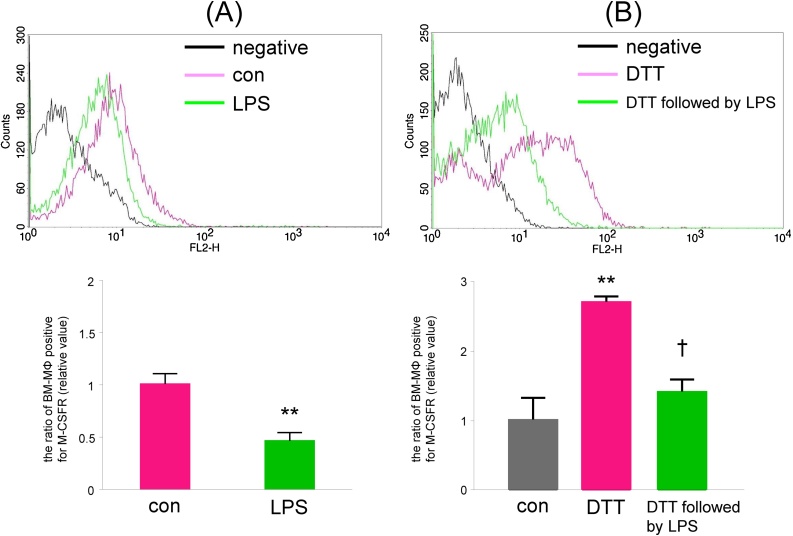
DTT-treated BM-MΦ can cause the downregulation of M-CSFR. BM-cultured cells were treated with vehicle (A) or DTT (1 mM, B) for 24 h followed by treatment with lipopolysaccharide (LPS, 2 μg/mL) for 24 h. The cells were then incubated with 400 ng/mL of Dio-conjugated Ac-LDL for 4 h, stained with PE-conjugated anti-M-CSFR antibody, and analyzed by flow cytometry. (Upper) Representative histograms for BM-MΦ (bone marrow cells that took up Ac-LDL) treated with LPS are shown. (Bottom) The proportion of BM-MΦ that was positive for M-CSFR (relative value) is shown. (A) n = 4, (B) n = 3, ** P < 0.01 vs. control (vehicle), † P < 0.05 vs. DTT-treated group.

It has been previously reported that lipopolysaccharide (LPS) downregulates M-CSFR [7,8]. We have also observed, as shown in Fig. 5A, that treatment with 2 μg/mL of LPS for 24 h decreased the level of M-CSFR on the cytoplasmic membrane (vehicle control; 1.0 ± 0.1, 24 h of LPS; 0.46 ± 0.08-fold). Additionally, treatment with 2 μg/mL of LPS for 24 h decreased the proportion of DTT-treated BM-MΦ that were positive for M-CSFR (Fig. 5B, control; 1.0 ± 0.3, DTT-treated group; 2.7 ± 0.1, 24 h of LPS after DTT-treatment; 1.4 ± 0.2-fold). These data suggest that DTT treatment increases the proportion of BM-MΦ that are positive for M-CSFR and that the upregulation of M-CSFR on BM-MΦ can be abrogated by subsequent treatment with LPS. Here, we propose a simple method to increase the number of M-CSFR-positive BM-MΦ using the reducing agent DTT.

## Additional information

The study conformed to the guidelines given in the Guide for the Care and Use of Laboratory Animals published by the US National Institutes of Health. The experimental protocol was approved by the Animal Care and Use Committee of Juntendo University.

Background: Macrophages play a role in the pathogenesis of atherosclerosis [9,10]. M-CSF (CSF-1) regulates monocyte/macrophage survival, proliferation, differentiation, and migration via the activation of its receptor (M-CSFR) [[11], [12], [13]]. Studies have demonstrated a decrease in aortic atherosclerosis in both M-CSF- /-/ApoE- /- [14] and M-CSF- /-/LDLR- /- [15] mice. It has also been reported that an M-CSFR-neutralizing antibody resulted in both a decrease in atherosclerosis [16] and pharmacologic inhibition of M-CSF signaling via a specific inhibitor of M-CSFR (GW2580), which also resulted in a decrease in atherosclerosis [17,18]. In this manner, the contributions of M-CSF and M-CSFR to atherogenesis, as well as the underlying mechanisms that are involved, have been revealed.

It has been reported that treatment with M-CSF, phorbol ester (12-O-tetradecanoylphorbol-13-acetate (TPA)), and LPS downregulates M-CSFR expression in macrophages [[19], [20], [21], [22], [23], [24]]; however, few studies of the relationship between the downregulation of M-CSFR and atherosclerosis have been reported. One of the contributing factors is the small proportion of macrophages that is positive for M-CSFR on the plasma membrane [1]. Thus, there is a need for a method to increase the number of macrophages that are positive for M-CSFR and investigate the mechanisms underlying the downregulation of M-CSFR during atherogenesis. In the present study, we have shown that treatment of BM-MΦ with DTT increased the proportion that was positive for M-CSFR and that the upregulation of M-CSFR on BM-MΦ can be abrogated by treatment with LPS. Here, we propose a simple method to increase the number of M-CSFR-positive BM-MΦ that utilizes the reducing agent DTT that may be useful in the investigation of the relationship between the downregulation of M-CSFR and some diseases such as atherosclerosis.

Future perspectives to reveal the relationship between the downregulation of M-CSFR and various diseases: We propose that DTT could be successfully utilized to investigate the relationship between the downregulation of M-CSFR and atherosclerosis. However, when using DTT in animal models in vivo, any possible side effects of DTT should be considered. For example, we may need to consider any cytoprotective effects that DTT may exert via an increase in the production of hydrogen sulfide (H_2_S) because there is now an abundance of scientific evidence that suggests that, despite its reputation as a noxious gas with wide-ranging cytotoxic effects, H_2_S, in fact, has cytoprotective effects [25].

H_2_S also protects neurons from oxidative stress by restoring the levels of glutathione, a major intracellular antioxidant, via enhancement of the activity of γ-glutamylcysteine synthetase and the transport of cysteine and cystine [26,27]. Additionally, it protects cardiomyocytes from ischemia/reperfusion injury by contributing to the preservation of mitochondrial function [28]. The production of H_2_S in mammalian systems has been attributed to three key enzymes: cystathionine β-synthase (CBS), cystathionine γ-lyase (CSE) and 3-mercaptopyruvate sulfurtransferase (3MST) [[29], [30], [31]]. 3MST produces H_2_S from 3-mercaptopyruvate (3 M P), which is generated by cysteine aminotransferase (CAT) from cysteine and α-oxoglutarate (α-KG) [[31], [32], [33], [34]]. 3 M P provides sulfur to the active-site cysteine residue of 3MST to produce persulfide, which, in turn, releases H_2_S in the presence of DTT [31,35]. Thus, the use of DTT, with due consideration to its possible cytoprotective effects, may facilitate the investigation of the relationship between the downregulation of M-CSFR and various diseases, including atherosclerosis, during future studies.

Discrepancy of the M-CSFR expression between this report and previous report: It was reported that 65.4 ± 3.0% of BM-cultured MΦ express M-CSFR [1], and we showed that 24.4 ± 4.6% of BM-cultured MΦ express M-CSFR. It was also reported that 3.6 ± 0.2% of peritoneal cultured macrophages express M-CSFR [1], and we showed that 95.3 ± 0.8% of isolated peritoneal macrophages express M-CSFR in this report. M-CSF, phorbol ester, and LPS are known to downregulate M-CSFR expression in macrophages [[19], [20], [21], [22], [23], [24]]. Thus, differences in the culture condition (especially serum differences) might cause the difference in M-CSFR expression in BM-cultured MΦ. The discrepancy in peritoneal macrophages is consistent with our data that the proportion of macrophages positive for M-CSFR is higher in isolated cells (37.8 ± 2.7% of isolated BM-MΦ; Fig. 1) than in cultured cells (24.4 ± 4.6% of BM-cultured MΦ; Fig. 2). Thus, the discrepancy in peritoneal macrophages might be explained by the difference between cultured cells and isolated cells.
